# Evaluation of the optimal dosage of S-1 in adjuvant SOX chemotherapy for gastric cancer

**DOI:** 10.3892/ol.2014.2821

**Published:** 2014-12-22

**Authors:** LIN YANG, YI YANG, QIONG QIN, AIPING ZHOU, JIANJUN ZHAO, JINWAN WANG, CHANG SHU, XINGHUA YUAN, SONGNIAN HU

**Affiliations:** 1 Chinese Academy of Medical Sciences, Cancer Institute and Hospital, Beijing 100021, P.R. China; 2 Chinese Academy of Sciences, Beijing Institute of Genomics, Beijing 100101, P.R. China

**Keywords:** adjuvant chemotherapy, gastric cancer, S-1, oxaliplatin

## Abstract

Gastric cancer (GC) is the second leading cause of cancer-related mortality worldwide. The usual treatment of GC consists of surgery with additional adjuvant chemotherapy. In the present study, the feasibility and safety of adjuvant S-1 plus oxaliplatin (SOX) chemotherapy for patients with GC and the optimal dosage of S-1 were determined. Eligible patients were randomly assigned to either arm A (30 cases) receiving 70 mg/m^2^ S-1 (in two seperate half doses) daily or arm B (30 cases) receiving 80 mg/m^2^ S-1 (in two seperate half doses) daily. The S-1 was administered twice daily for 14 days followed by a 7-day rest period for the third week. A total of 130 mg/m^2^ oxaliplatin was administered on day 1 every 3 weeks for each arm. The cumulative rates of the relative total administration dose of S-1 at 100% in the 6th treatment course was 71.4% [95% confidence interval (CI), 56.5–90.3%] in arm A, which was significantly higher than 21.4% (95% CI, 10.5–43.6%) in arm B (P=0.001). The most common grade 3/4 toxicities were neutropenia (19.6%), thrombocytopenia (19.6%) and vomiting (16.1%). Grade 3/4 thrombocytopenia was observed in 7.1% of patients in arm A and in 32.1% of patients in arm B (P=0.019). With regard to the adverse events induced by S-1 administration, the incidence of diarrhea (3.6 vs. 42.9%; P<0.001) was significantly higher in arm B than in arm A, as anticipated. Collectively, adjuvant SOX therapy for GC is feasible and safe, and when combined with 130 mg/m^2^ oxaliplatin, 70 mg/m^2^/day S-1 appears to the optimal dose.

## Introduction

Gastric cancer (GC) is the second most common cause of cancer-related mortality worldwide and the third most common cancer in China (1,2). The primary treatment for operable GC is surgery. However, recurrence rates are high when using surgery only (3,4). Compared with surgery only, additional adjuvant chemotherapy has shown clinical benefits in treating GC when evaluated by meta-analyses (5,6). Therefore, it is necessary to develop optimal adjuvant chemotherapy regimens to decrease recurrence and improve the quality of life for GC patients following surgical resection.

S-1 is a fourth generation oral fluoropyrimidine, which contains tegafur/gimeracil/oteracil potassium in a molar ratio of 1.0:0.4:1.0. Treatment with S-1 subsequent to surgery has been shown to improve the 5 year overall survival (OS) rate of GC patients from 61.1% with surgery alone to 71.1%, and the relapse-free survival rate of GC patients in 5 years from 53.1 to 65.4% (7). However, a subgroup data analysis showed that use of adjuvant chemotherapy with S-1 alone following surgery for patients diagnosed with stage III GC did not result in improved survival (7).

For metastatic or recurrent GC, adjuvant chemotherapy with S-1 plus cisplatin (SP) showed improved results, with a longer progression-free survival (PFS) time and a longer OS time, compared with S-1 alone (8). Compared with cisplatin, oxaliplatin has a more favorable safety profile, including less emetogenic and less nephrotoxic potential. A REAL-2 study revealed that a oxaliplatin-based regimen was just as effective as a cisplatin-based regimen in patients with previously untreated advanced GC (AGC) (9). Additionally, a large randomized phase III study recently reported that S-1 plus oxaliplatin (SOX) showed non-inferiority to SP in PFS and that the treatment was well tolerated, with benefits in terms of outpatient-based treatment in patients with AGC (10).

In our previous study, the SOX regimen with 130 mg/m^2^ oxaliplatin was found to be effective and safe to use as a first-line chemotherapy in patients with AGC (11). Therefore, the dose of oxaliplatin was fixed at 130 mg/m^2^ in the current study. A dose of S-1 at 80 mg/m^2^ (in two seperate half doses) twice daily on days 1 to 14 every 21 days is widely used for phase II/III studies (12,13). However, in our previous dose-finding study on adjuvant chemotherapy with SOX (130 mg/m^2^ oxaliplatin) for GC, the maximum tolerated dose of S-1 was initially determined to be 70 mg/m^2^ (14). Grade 3 vomiting was observed as dose-limiting toxicity (DLT) during the first treatment cycle in this study, highlighting the fact that oxaliplatin may play an important role in DLT. Based on these studies, we hypothesized that from the two doses (70 vs. 80 mg/m^2^/day) there should be an optimal dosage of S-1 when combined with oxaliplatin (130 mg/m^2^) in adjuvant chemotherapy for GC. The aim of the present study was therefore to evaluate the feasibility and safety of adjuvant SOX chemotherapy for GC patients. In addition, the present study aimed to determine the optimal dosage of S-1 in the SOX combination for GC patients.

## Patients and methods

### Patients

A total of 60 eligible patients at the Cancer Institute and Hospital of the Chinese Academy of Medical Sciences (Beijing, China) were recruited for this study according to the following inclusion criteria: Subtotal or total gastrectomy; histologically proven stage II/III [i.e., pathological stage T2N+, T3–T4 and/or N+, according to the American Joint Committee on Cancer tumor-node-metastasis system, 7th edition (15)] GC of the stomach or gastroesophageal junction; age distribution of 20 to 75 years old; an Eastern Cooperative Oncology Group performance status (16) of 0–1; an absolute granulocyte count of >1,500/l; a platelet count of >100,000/l; a hemoglobin level of >90 g/l; a serum bilirubin level of less than the upper limit of normal (ULN); a normal creatinine level; an alanine transaminase and aspartate transaminase level of <1.5×ULN; and no treatment with chemotherapy prior to the present SOX treatment. Only patients who could swallow tablets were admitted into the study group, and all patients were told to practice medically effective contraception.

### Study approval and consent

The present study was approved by the Ethics Committee of the Cancer Institute and Hospital, (Chinese Academy of Medical Sciences, Beijing, China). All patients provided written informed consent.

### Treatment schedule

Treatment with SOX therapy was started at 4–8 weeks post-surgery, and repeated for 8 cycles. All eligible patients were randomly assigned to either arm A receiving S-1 at a dose of 70 mg/m^2^/day (in two seperate half doses) or arm B receiving S-1 at a dose of 80 mg/m^2^/day (in two seperate half doses). S-1 was administered orally twice per day, within half an hour of a meal on days 1 to 14, every 3 weeks (1 cycle). Oxaliplatin was administered intravenously to all patients on day 1 every 3 weeks at a fixed dose of 130 mg/m^2^. All patients received 5-HT3 antagonists as antiemetics following administration of oxaliplatin. If patients developed grade 4 neutropenia or grade 3/4 thrombocytopenia, or non-hematological toxic effects of above grade 2, the dose of S-1 was reduced by 10 mg/m^2^/day, and at the same time, the dose of oxaliplatin was reduced by 25%. If recovery from such toxicities was confirmed at a reduced dose, the administration at the reduced dosage was continued. S-1 and oxaliplatin could be reduced twice, but treatment was discontinued if subsequent reduction was indicated. In cases of oxaliplatin-related neurological adverse events, S-1 could be continued as monotherapy. Oxaliplatin monotherapy was not allowed if S-1 was discontinued.

Complete blood count and blood chemistry studies were performed weekly. Administration of the two agents would be delayed until adequate hematological recovery (absolute neutrophil count, ≥1.5×10^9^/l; platelet count, ≥100×10^9^/l) was achieved. Non-hematological toxicities, excluding alopecia, were required to be grade 1 or better prior to initiation of each cycle. If the toxicity failed to recover within 3 weeks after the scheduled day for starting the next cycle, the patients were withdrawn from the study. Therapy was discontinued if there was any evidence of documented recurrence, unacceptable toxicities or refused treatment.

### Evaluation

All eligible patients were considered to be assessable for feasibility and safety. Adverse events were graded according to the National Cancer Institute-Common Toxicity Criteria version 4.0 (17). Feasibility was evaluated by the completion status of the protocol treatment and compared between the two arms. The rate of completing ≥6 and 8 cycles of treatment, delayed courses and dose reduction was evaluated and compared in the two arms. The number of patients was calculated at the time when the treatment was stopped or delayed, and when the planned administration dose was reduced. In the two arms, the relative total administration dose in the 6th treatment course (R6) and the 8th treatment course (R8) were calculated as follows: R6 = (D1 + D2 + D3 + D4 + D5 + D6) / (P1 × 6) and R8 = (D1 + D2 + D3 + D4 + D5 + D6 + D7 + D8) / (P1 × 8), where R is the relative total administration dose, D is the actual dose in each cycle and P1 is the planned dose in the first cycle. If no treatment was offered in this cycle, the actual dose was set to zero. The cumulative rate of the relative total administration dose in the 6th and 8th treatment courses were also calculated and compared in two arms.

### Statistics

Patient characteristics, feasibility and adverse events were analyzed. The differences in the median body surface area and ages between the two arms were evaluated by the Mann-Whitney U test, while other characteristics were evaluated by the χ^2^ test. The differences in completion status of protocol treatment and adverse events between the two arms were evaluated by the χ^2^ test. The differences in the relative total administration dose between the two arms were compared by Student’s t-test. The cumulative rates of the relative total administration dose of S-1 and oxaliplatin were examined by the Kaplan-Meier method and differences in the two arms were calculated by the log-rank test. Two-sided P<0.05 was used to indicate a statistically significant difference.

## Results

### Study population

**Figure 1:**
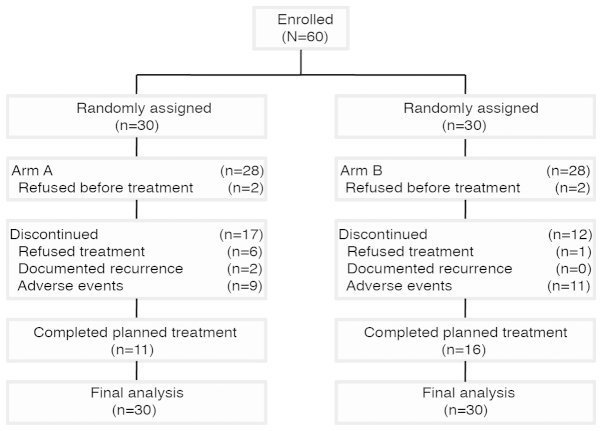
CONSORT (18) diagram showing patient disposition. Arm A, 70 mg/m^2^/day S-1; arm B, 80 mg/m^2^/day S-1.

Between June 2011 and June 2013, 60 patients were recruited to the study. Among these, two patients in arm A and two patients in arm B refused treatment and were excluded from all analyses. Patient disposition throughout the study is shown in Fig. 1. The major reasons for discontinuation of treatment in arm A were documented recurrence (6.7%), adverse events (30%) and refused treatment (20%). However, notably, four patients refused treatment subsequent to finishing 6 cycles of therapy in arm A. Patient baseline characteristics were well balanced between the two arms (Table I).

### Feasibility

In total, 48.2% of patients completed the planned 8 cycles of treatment and 75% of patients completed 6 cycles of therapy. A total of 39.3% of patients in arm A received the full 8 cycles of SOX compared with 57.1% in arm B [not significant (NS), P=0.181] (Table II). However, 82.1% of patients in arm A received ≥6 cycles of therapy, which was higher the percentage of 67.9% in arm B (NS, P=0.217). In total, 91.1% of patients were observed with delayed courses. Among them, 82.1% of patients underwent delayed courses in arm A, while 100% were observed in arm B (P=0.019). In total, the rate of patients with a dose-reduction of S-1 and oxaliplatin was 33.9% and 42.9%, respectively. The rate of patients with a dose-reduction of S-1 was 14.3% in arm A and 53.6% in arm B (P=0.002). In the patients with a dose-reduction of oxaliplatin, a rate of 39.3% was observed in arm A and 46.4% was observed in arm B (NS, P=0.558).

The mean of the relative total administration dose of S-1 in the 6th treatment course was 89.43% in arm A and 81.36% in arm B (NS, P=0.213), and the mean of the relative total administration dose of S-1 in the 8th treatment course in arm A was higher than that in arm B (77.18 vs. 73.07%; NS, P=0.551) (Table III). The mean of the relative total administration dose of oxaliplatin in the 6th treatment course was 83.57% in arm A and 81.89% in arm B (NS, P=0.810), and the mean of the relative total administration dose of oxaliplatin in the 8th treatment course in arm A was higher that in arm B (66.57 vs. 70.68%; NS, P=0.540) (Table III).

**Figure 2:**
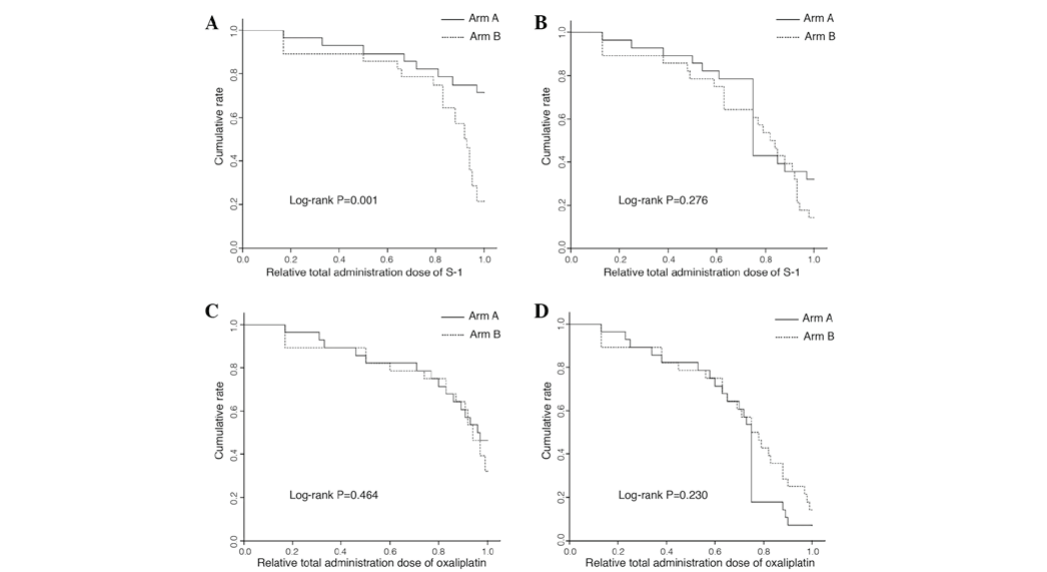
Kaplan-Meier estimation of cumulative rate of relative total administration dose among 56 gastric cancer patients. (A) Cumulative rate of relative total administration dose of S-1 in the 6th treatment course in each arm. (B) Cumulative rate of relative total administration dose of S-1 in the 8th treatment course in each arm. (C) Cumulative rate of relative total administration dose of oxaliplatin in the 6th treatment course in each arm. (D) Cumulative rate of relative total administration dose of oxaliplatin in the 8th treatment course in each arm.

The cumulative rates of the relative total administration dose of S-1 in the 6th treatment course at 100% was 71.4% (95% CI, 56.5–90.3%) in arm A, which was significantly higher than the 21.4% (95% CI, 10.5–43.6%) in arm B (P=0.001) (Fig. 2A; Table III). However, when calculated in the 8th treatment course, the rates were 32.1% (95% CI, 18.8–55.1%) in arm A and 14.3% (95% CI, 5.77–35.4%) in arm B (NS, P=0.276) (Fig. 2B; Table III). The cumulative rates of the relative total administration dose of oxaliplatin in the 6th treatment course at 100% were 46.4% (95% CI, 31.2–69.1%) in arm A and 32.1% (95% CI, 18.8–55.1%) in arm B (NS, P=0.464) (Fig. 2C; Table III). Additionally, when calculated in the 8th treatment course, the rates were 7.14% (95% CI, 1.88–27.2%) in arm A and 14.3% (95% CI, 5.77–35.4%) in arm B (NS, P=0.23) (Fig. 2D; Table III).

### Adverse events

Drug-related adverse events are listed in Table IV. In total, the most common grade 3/4 hematological toxicities were neutropenia (19.6%) and thrombocytopenia (19.6%). A total of 10.7% of patients in arm A and 28.6% in arm B experienced grade 3/4 neutropenia (NS, P=0.093), while grade 3/4 thrombocytopenia was observed in 7.1% of patients in arm A and in 32.1% of patients in arm B (P=0.019). Grade 1/2 thrombocytopenia was observed at different frequencies in each arm, with 15 patients (53.6%) in arm A and 8 patients (28.6%) in arm B (NS, P=0.057). Only one patient in arm B and no patients in arm A developed grade 3 anemia (NS, P=0.313).

In total, the most common grade 3/4 non-hematological toxicity was vomiting (16.1%). With regard to the overall incidence of adverse events, hyperpigmentation, asthenia, nausea and neurotoxicity were the most frequent non-hematological toxicities in each arm. Nausea, with the highest rates of grade 1/2 non-hematological toxicity, was observed in 71.4% of patients in arm A and 89.3% in arm B (NS, P=0.093). With regard to the adverse events induced by S-1 administration, the incidence of diarrhea (3.6 vs. 42.9%; P<0.001) was significantly higher in arm B compared with arm A, as anticipated.

Thrombocytopenia was the most frequent reason for dose reduction and cycle delay in each arm. Among the non-hematological toxicities, vomiting in arm A and diarrhea in arm B were the most common reasons for dose reduction and cycle delay.

## Discussion

The role of post-operative adjuvant chemotherapy following curative D2 gastrectomy has long been debated. Multiple randomized, controlled studies have evaluated the role of post-operative adjuvant chemotherapy for GC (19–21), however, as a result of population and regimen heterogeneity, no consensus has been reached with regard to the chemotherapeutic regimen, schedule or duration of adjuvant chemotherapy. The results of the G-SOX study reflected the efficacy and safety of SOX in patients with AGC (12). Therefore, SOX is considered to be a candidate for an experimental arm in the next adjuvant chemotherapy trial.

In the present study, 48.2% of patients completed 8 cycles as planned, however, 75% of patients received >6 cycles of treatment. SOX has shown more advantages compared with the SP regimen in adjuvant chemotherapy for GC patients, since 22.6% completed 5 cycles as planned in the CCOG0703 study (22) and 60.8% completed 6 cycles as planned in the study by Kang *et al* (23). In the present study, the treatments were generally well tolerated. The most frequently observed grade 3/4 toxicities were neutropenia (19.6%), thrombocytopenia (19.6%) and vomiting (16.1%).

To the best of our knowledge, this is the first randomized feasibility study comparing two common doses of S-1 in combination with oxaliplatin in GC patients following curative surgery. From the viewpoint of the completion status of the protocol treatment, the completion rate was not significantly different, however, significantly less delay to the course was observed in arm A (91.1 vs. 82.1%; P=0.019). Furthermore, less grade 3/4 neutropenia (19.6 vs. 10.7%; P=0.093) and thrombocytopenia (grade 1/2, 41.1 vs. 53.6%; NS, P=0.057; and grade 3/4, 19.6 vs. 7.1%; P=0.019) occurred in arm A, which may have contributed to the less delay to the course. With regard to the adverse events induced by S-1 administration, the incidence of diarrhea (3.6 vs. 42.9%; P<0.001) was significantly higher in arm B than in arm A, as anticipated.

The rate of discontinued cases was higher in arm A than in arm B, however, notably, four patients refused treatment subsequent to finishing 6 cycles of therapy in arm A for grade 1 asthenia. The cumulative rate of the relative total administration dose of S-1 in the 6th treatment course at 100% was 71.4% (95% CI, 56.5–90.3%) in arm A, which was significantly higher than the 21.4% (95% CI, 10.5–43.6%) in arm B (P=0.001). Additionally, when calculated in the 8th treatment course, the rate was 32.1% (95% CI, 18.8–55.1%) in arm A, which was higher than the 14.3% (95% CI: 5.77–35.4%) in arm B (NS, P=0.276). The relative total administration dose of S-1 in the 6th treatment course and the 8th treatment course were higher in arm A than arm B. However, no significant difference in the administration dose of oxaliplatin was found between the two arms.

These results suggested that a regimen using S-1 at a dose of 70 mg/m^2^ twice daily for 14 days followed by a 7-day rest period is more acceptable compared with the regimen of S-1 at 80 mg/m^2^ twice daily for 14 days, when combined with oxaliplatin at 130 mg/m^2^ on day 1 every 3 weeks. Owing to the lack of a survival analysis and the small sample size in the current study, the potential to reduce relapse following treatment in GC patients post-surgery should be carefully examined in the future. Patients in the present study will continue to be observed for recurrence and survival.

## Figures and Tables

**Table I:** Patient characteristics.

	Arm A (n=30)		Arm B (n=30)		Total (n=60)	
			
Characteristics	No.	%	No.	%	No.	%
Age, years						
Median	53.5		52.5		53	
Range	28–72		27–67		27–72	
BSA, m^2^						
Median	1.705		1.705		1.705	
Range	1.35–2.01		1.48–1.98		1.35–2.01	
Gender						
Male	24	80.0	23	76.7	47	78.3
Female	6	20.0	7	23.3	13	21.7
ECOS PS						
0	13	43.3	13	43.3	26	43.3
1	17	56.7	17	56.7	34	56.7
Type of gastrectomy						
Total	6	20.0	5	16.7	11	18.3
Partial	24	80.0	25	83.3	49	81.7
TNM stage						
IA	2	6.7	2	6.7	4	6.7
II	9	30.0	8	26.7	17	28.3
III	18	60.0	19	63.3	37	61.7
IV	1	3.3	1	3.3	2	3.3

ECOS PS, Eastern Cooperative Oncology Group performance status; BSA, body surface area; TNM, tumor-node-metastasis.

**Table II:** Completion status of protocol treatment.

	Total (n=56)		Arm A (n=28)		Arm B (n= 28)		
				
	No.	%	No.	%	No.	%	P-value
Patients received ≥6 cycles of therapy	42	75.0	23	82.1	19	67.9	0.217
Patients received 8 cycles of therapy	27	48.2	11	39.3	16	57.1	0.181
Patients with delayed courses	51	91.1	23	82.1	28	100.0	0.019
Patients with dose-reduction							
S-1	19	33.9	4	14.3	15	53.6	0.002
Oxaliplatin	24	42.9	11	39.3	13	46.4	0.558

**Table III:** Relative administration dose analysis of S-1 and oxaliplatin.

Parameter	Arm A (n=28)	Arm B (n=28)	P-value
Cumulative rate of relative total administration dose of S-1 at 100% in the 6th treatment course, % (95% CI)	71.40 (56.50–90.30)	21.40 (10.50–43.60)	0.001
Relative total administration dose of S-1 (6th treatment course), %			0.213
Mean	89.43	81.36	
Standard deviation	22.08	25.70	
Cumulative rate of relative total administration dose of S-1 at 100% in the 8th treatment course, % (95% CI)	32.10 (18.80–55.10)	14.30 (5.77–35.40)	0.276
Relative total administration dose of S-1 (8th treatment course), %			0.551
Mean	77.18	73.07	
Standard deviation	23.74	27.36	
Cumulative rate of relative total administration dose of OXA at 100% in the 6th treatment course, % (95% CI)	46.40 (31.20–69.10)	32.10 (18.80–55.10)	0.464
Relative total administration dose of OXA (6th treatment course), %			0.810
Mean	83.57	81.89	
Standard deviation	24.74	27.09	
Cumulative rate of relative total administration dose of OXA at 100% in the 8th treatment course, % (95% CI)	7.14 (1.88–27.20)	14.30 (5.77–35.40)	0.230
Relative total administration dose of OXA (8th treatment course), %			0.540
Mean	66.57	70.68	
Standard deviation	22.13	27.38	

OXA, oxaliplatin; CI, confidence interval.

**Table IV:** Drug-related adverse events.

	Total (n=56)		Arm A (n=28)		Arm B (n=28)			
				
	G1/2	G3/4	G1/2	G3/4	G1/2	G3/4	P-value	
				
Toxicity^a^	No. (%)	No. (%)	No. (%)	No. (%)	No. (%)	No. (%)	G1/2	G3/4
Anemia	20 (35.7)	1 (1.8)	10 (35.7)	0 (0.0)	10 (35.7)	1 (3.6)	1.000	0.313
Leukopenia	39 (69.6)	4 (7.1)	20 (71.4)	1 (3.6)	19 (67.9)	3 (10.7)	0.771	0.299
Neutropenia	32 (57.1)	11 (19.6)	18 (64.3)	3 (10.7)	14 (50.0)	8 (28.6)	0.280	0.093
Thrombocytopenia	23 (41.1)	11 (19.6)	15 (53.6)	2 (7.1)	8 (28.6)	9 (32.1)	0.057	0.019
TBIL	2 (3.6)	0 (0.0)	2 (7.1)	0 (0.0)	0 (0.0)	0 (0.0)	0.150	-
Hyperpigmentation	33 (58.9)	0 (0.0)	18 (64.3)	0 (0.0)	15 (53.6)	0 (0.0)	0.415	-
Asthenia	37 (66.1)	0 (0.0)	17 (60.7)	0 (0.0)	20 (71.4)	0 (0.0)	0.397	-
Nausea	45 (80.4)	4 (7.1)	20 (71.4)	2 (7.1)	25 (89.3)	2 (7.1)	0.093	1.000
Vomiting	25 (44.6)	9 (16.1)	12 (42.9)	4 (14.3)	13 (46.4)	5 (17.9)	0.788	0.716
Stomatitis	4 (7.1)	0 (0.0)	2 (7.1)	0 (0.0)	2 (7.1)	0 (0.0)	1.000	-
Diarrhea	13 (23.2)	0 (0.0)	1 (3.6)	0 (0.0)	12 (42.9)	0 (0.0)	<0.001	-
Neurotoxicity	42 (75.0)	0 (0.0)	22 (78.6)	0 (0.0)	20 (71.4)	0 (0.0)	0.537	-
Hand-foot syndrome	0 (0.0)	0 (0.0)	0 (0.0)	0 (0.0)	0 (0.0)	0 (0.0)	-	-
ALT elevation	13 (23.2)	0 (0.0)	6 (21.4)	0 (0.0)	7 (25.0)	0 (0.0)	0.752	-

G, grade; TBIL, total bilirubin; ALT, alanine transaminase.

a If a patient had multiple occurrences of the same adverse event, it was counted with the highest grade.
